# Tailoring Cu Nanoparticle Catalyst for Methanol Synthesis Using the Spinning Disk Reactor

**DOI:** 10.3390/ma11010154

**Published:** 2018-01-17

**Authors:** Christian Ahoba-Sam, Kamelia V. K. Boodhoo, Unni Olsbye, Klaus-Joachim Jens

**Affiliations:** 1 Department of Process, Energy and Environmental Technology, University College of Southeast Norway, Kjølnes Ring 56, 3918 Porsgrunn, Norway; christian.ahoba-sam@usn.no; 2 School of Engineering, Merz Court, Newcastle University, Newcastle Upon Tyne NE1 7RU, UK; kamelia.boodhoo@newcastle.ac.uk; 3 Department of Chemistry, University of Oslo, P.O. Box 1033, Blindern, N-0315 Oslo, Norway; unni.olsbye@kjemi.uio.no

**Keywords:** Cu nanoparticles, spinning disc reactor, methanol synthesis, low temperature

## Abstract

Cu nanoparticles are known to be very active for methanol (MeOH) synthesis at relatively low temperatures, such that smaller particle sizes yield better MeOH productivity. We aimed to control Cu nanoparticle (NP) size and size distribution for catalysing MeOH synthesis, by using the spinning disk reactor. The spinning disk reactor (SDR), which operates based on shear effect and plug flow in thin films, can be used to rapidly micro-mix reactants in order to control nucleation and particle growth for uniform particle size distribution. This could be achieved by varying both physical and chemical operation conditions in a precipitation reaction on the SDR. We have used the SDR for a Cu borohydride reduction to vary Cu NP size from 3 nm to about 55 nm. XRD and TEM characterization confirmed the presence of Cu_2_O and Cu crystallites when the samples were dried. This technique is readily scalable for Cu NP production by processing continuously over a longer duration than the small-scale tests. However, separation of the nanoparticles from solution posed a challenge as the suspension hardly settled. The Cu NPs produced were tested to be active catalyst for MeOH synthesis at low temperature and MeOH productivity increased with decreasing particle size.

## 1. Introduction

Methanol (MeOH) is a multi-purpose molecule widely used as a base chemical, for energy, and CO_2_ storage [1]. It is used as a solvent or as an intermediate for the production of formaldehyde, methyl tert-butyl ether, acetic acid, methyl methacrylate, and other fine chemicals. MeOH can also be used as fuel blend or directly converted to valuable hydrocarbons such as gasoline over acidic microporous materials [2], thereby providing alternative sources of petrochemical feedstock used today.

Currently, the technology for MeOH synthesis is based on conversion of synthesis gas (composed largely of 2H_2_/CO with about 5% CO_2_) over CuO/ZnO/Al_2_O_3_ catalyst operating at around 250–300 °C and 50–100 bar [3]. Even though this process is highly optimized, the thermodynamics of the reaction limit syngas conversion per pass, which, coupled with other operational costs, make the process capital intensive. For example, more than 60% of the total capital cost in current MeOH processes is associated with the syngas plant [4].The lowest cost of syngas production is by the use of air rather than a pure cryogenic O_2_-blown autothermic reformer [3]. Syngas conversion to MeOH is highly exothermic (shown in Equation (1)) and lower temperatures are required to achieve full conversion per pass. A full conversion per pass process allows the use of N_2_ diluted syngas for MeOH production, which implies no need for recycling of unconverted reactants. Consequently, the carbon footprint is reduced [5] as a result of the full conversion.

(1) $$CO+2H_{2}⇌CH_{3}OH\;\Delta H=-90.6\;\mathrm{kJ}/\mathrm{mol}$$

Alternatively, low temperature MeOH synthesis (LTMS), which proceeds rapidly in liquid medium at about 100 °C presents the possibility for full syngas conversion per pass [6]. This technology is known to occur in two steps as shown in Equations (2) and (3). The carbonylation of MeOH to methyl formate (Equation (2)) is catalysed by an alkali alkoxide, while a transition metal based compound catalyses the hydrogenolysis of methyl formate (Equation (3)).

(2) $$CO+CH_{3}OH⇌HCOOCH_{3}\;$$

(3) $$\;\;\;\;\;HCOOCH_{3}+2H_{2}⇌2CH_{3}OH\;$$

The Cu-based catalyst is one catalyst which has received a lot of attention for LTMS [7,8,9,10,11]. Examples of Cu-based materials reported for the hydrogenolysis reaction include CuO/Cr_2_O_3_, Raney Cu, Cu on SiO_2_, CuCl_2_, and Cu alkoxide. Prolonged milling of a physical mixture of CuO and Cr_2_O_3_, for example, enhanced MeOH synthesis activity [7]. We have also observed that Cu nanoparticle (NP) sizes influenced MeOH production, such that MeOH productivity increased with decreasing the particle size [10]. In both instances, MeOH productivity correlated well with increasing total surface area. This implies that producing the right-sized Cu particles as a catalyst for MeOH synthesis is important. In general, different Cu NPs sizes can be synthesized by following different experimental protocols and recipes [12]. Based on MeOH yield dependence on the Cu NP sizes, an on-purpose physical method for making Cu NP catalyst of different sizes using a specific chemical recipe will be a valuable contribution. In this work, we will focus on the use of spinning disk reactor (SDR), a technique that can fine-tune the Cu NP catalyst size for MeOH synthesis. 

The SDR is a continuous-flow process intensification reactor with enhanced production efficiency, safety, minimal cost, and minimal waste technology [13,14]. A thin film liquid is formed in the SDR due to centrifugal acceleration created by rotation of the disk. The key characteristics of the thin film flow include rapid mixing, heat and mass transfer, plug flow, and short residence times in the order of seconds [15]. For example, the residence time, *t_res_* of liquid reagents traveling with Q flow rate, from *r_i_* to *r_o_* on the disk based on the Nusselt theory can be expressed by Equation (4), where μ is dynamic viscosity and ω is angular velocity. Hence, increasing the flow rate and rotation speed for example will lead to a shorter residence time and consequently affect crystallization process.

(4) $$t_{res}=\frac{3}{4}{\left(\frac{12\pi^{2}\mu }{Q^{2}\omega^{2}}\right)}^{\frac{1}{3}}\left(r_{o}^{\frac{4}{3}}-r_{i}^{\frac{4}{3}}\right)$$

The SDR can therefore be employed in sol-gel precipitation processes where homogenous mixing of the reactants at the molecular level is essential for controlling crystallite and particle size. Recently, the SDR has been used in several precipitation reactions for nanoparticles production [13,16,17,18]. Tai et al. [17], for example, used the SDR to produce 40–50 nm CuO nanoparticles using Cu(SO_4_) and Na(CO_3_)_2_ as reactants for nanofluid application. 

In this work, we have used the SDR to produce different Cu NP sizes and size distributions, in a more environmentally friendly condition, using aqueous borohydride and Cu(NO_3_)_2_ as reactants. Our aim was to find out if the SDR could be used to purposefully produce Cu NP catalysts for MeOH synthesis. We found out that varying physical parameters of the SDR could fine-tune Cu NP catalyst sizes using specific chemical recipe. Furthermore, we scaled-up the Cu NP production for catalytic application in low temperature MeOH synthesis. 

## 2. Results and Discussion

**Figure 1:**
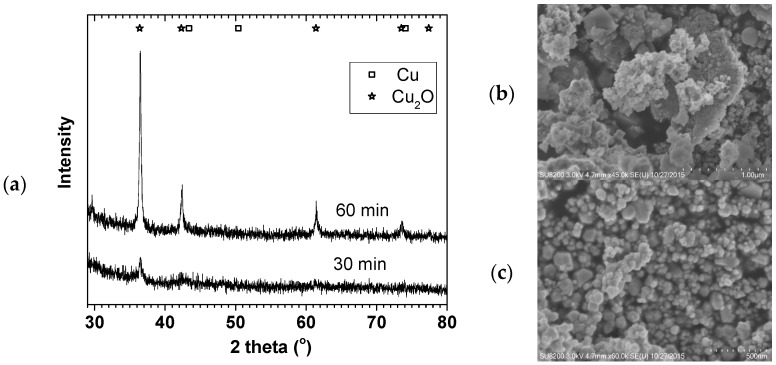
XRD (**a**) and SEM ((**b**) = 30 min & (**c**) = 60 min) of Cu NP made in a stirred tank; 0.011 M Cu(CH_3_COO)_2_ and 0.021 M NaBH_4_.

The SDR technique involves continuous flow of reactant and product stream with short residence time (in seconds) and therefore the process of producing fine nanoparticles must be a fast reaction. In view of this, our Cu particles were made using borohydride reduction (Equation (5)) [19] which occurs instantaneously when Cu^2+^ react with NaBH_4_. Our preliminary test in a flask stirred at 700 rpm showed that when 0.011 M Cu^2+^ solution was added dropwise to 0.021 M BH_4_^−^ solution, a black precipitation occurred instantaneously. Figure 1 shows the X-ray diffraction (XRD) and SEM images of 70 °C oven dried samples. Although Cu^0^ was the expected product, the oven drying in air easily leads to Cu^0^ surface oxidation. The XRD and SEM confirmed the formation of Cu_2_O with 9 ± 1 and 25 ± 1 nm crystallite sizes.

(5) $$Cu{\left(NO_{3}\right)}_{2}+2NaBH_{4}+6H_{2}O\to Cu↓+7H_{2}+2NaNO_{3}+2B{\left(OH\right)}_{3}\;$$

The following sections will however focus on the use of the SDR to control Cu particle size. Here, the Cu salt and borohydride solution comes into contact at a shorter and easily controllable residence time compared with the stirred tank approach described above. The resulting slurry from the SDR was collected in starch to avoid settling and agglomeration of the particles after collection [16,20]. The starch serves as a capping agent to stabilize the Cu NPs made as well as to prevent further growth of particles.

### 2.1. Effect of Rotation of Disk Speed and Flow Rate

**Figure 2:**
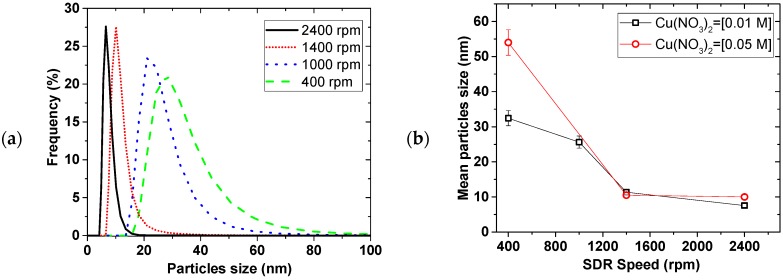
Effect of SDR spinning on (**a**) PSD for 0.01 M Cu(NO_3_)_2_ and (**b**) mean particle size, at 1.7 mL/s of 0.01 M and 0.05M Cu(NO_3_)_2_; 3.3 mL/s of 0.02 M and 0.10 M NaBH_4_ (in 0.004 M NaOH).

**Figure 3:**
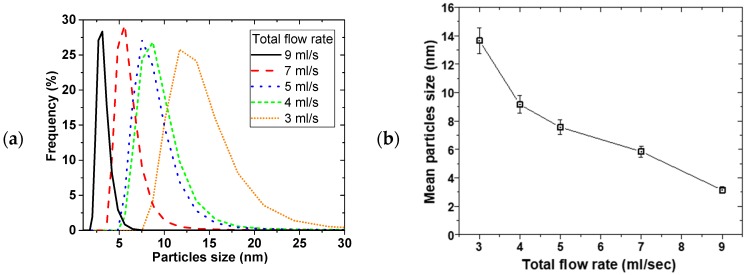
Effect of flow rate on (**a**) PSD and (**b**) mean particle size, 0.02 M NaBH_4_ (in 0.004 M NaOH)/0.01 M Cu(NO_3_)_2_ flow ratio = 2, disk speed = 2400 rpm.

Figure 2 shows the effect of the disk rotation speed on particle size distribution (PSD) for a 0.01 M Cu(NO_3_)_2_ and 0.02 M NaBH_4_ solution at 5 mL/s total flow rate. The faster the rotation, the narrower the PSD, while the mean particle sizes decreased from 35 ± 2 to 7.6 ± 0.5 nm for the 0.01 M Cu(NO_3_)_2_, with increasing disk speed from 400 to 2400 rpm, respectively. This trend was the same at both 0.01 and 0.05 M Cu(NO_3_)_2_ starting concentrations. Figure 3 shows the effect of the total flow rate on PSD, at constant 0.02 M NaBH_4_ flow to 0.01 M Cu(NO_3_)_2_ flow ratio of 2 and 2400 rpm disk rotation speed. Similarly, mean particle sizes decreased from 14 ± 1 to 3.2 ± 0.2 nm with narrowing PSD as the flow rate was increased from 3 to 9 mL/s, respectively.

The narrowing of particle size distribution with disk rotation speed and flow rate can be explained by the degree of micromixing achieved under the tested conditions. It is expected that shear effect of thin film formed on the disk and surface wave intensity both increase with increasing disk rotation speed and flow rate [13,14]. Mohammadi et al. [13] for example, showed that higher rotation speed and faster flow rate led to shorter micromixing time in the precipitation of titanium hydroxide. This results in more rapid homogeneous mixing at the molecular level coupled with more uniform supersaturation being attained. Short micromixing time favours nucleation over growth [21] leading to small particle formation. Uniformly sized nuclei lead to narrow particle size distribution as observed at high disk rotation speeds and faster flow rates.

The SDR mean residence time and its residence time distribution (RTD) also have a significant influence on the final particle size and PSD respectively [13,22,23,24]. Increasing the rotation speed and flow rate leads to shorter residence time of the nuclei formed on initial contact of the reactants on the disk, thereby limiting the extent of particle growth and agglomeration in the SDR. It is also well-established that under conditions of high disk speeds and high flowrate, the RTD of the film approaches a near plug flow profile [23]. Under a plug flow regime, practically all particles will be subjected to the same mean residence time and processing conditions due to minimal radial dispersion and will exit the disk with a uniform particle size, resulting in tight PSDs. Clearly, the beneficial effects of high disk speeds and reagent flowrates on the film hydrodynamics are wide ranging and have a considerable impact on the formation of Cu NPs in this work. The best operating conditions are found to be 2400 rpm and 9 mL/s total flow rate.

**Figure 4:**
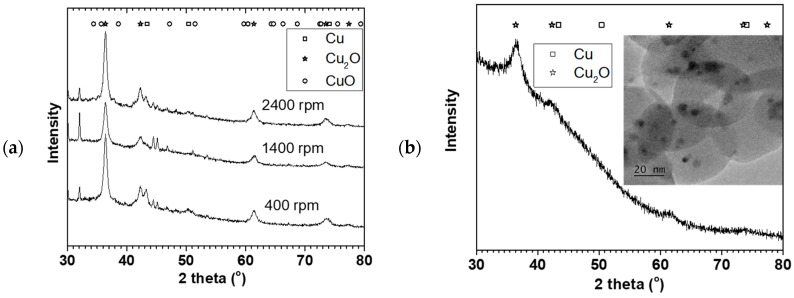
(**a**) XRD of the 0.05 M Cu precursor samples in Figure 2b, and (**b**) XRD with TEM image of the 9 mL/s sample in Figure 3.

Figure 4a shows X-ray diffraction of the NPs made at the 0.05 M Cu(NO_3_)_2_ starting concentration (in Figure 2) oven dried at 70 °C. The XRD showed predominantly Cu_2_O phase with some Cu phase (plus some NaNO_3_ reflections). The crystallite sizes estimated using the TOPAS software for the Cu_2_O phase were 10 ± 1, 9.5 ± 0.7, and 9.5 ± 0.5 nm for 400, 1400, and 2400 rpm respectively. The crystallite sizes were similar for the three rotations despite their particle sizes and distribution differences. Figure 4b shows the XRD and TEM image of the NP made at 9 mL/s flow rate and 2400 rpm disk speed (in Figure 3). Similarly, Cu_2_O phase was predominant with 4 ± 1 nm crystallite size. Furthermore, TEM image of the sample showed about 3–5 nm spherical shaped crystals surrounded by large amorphous materials likely to be the starch used to keep the particles from agglomerating. The XRD and TEM confirmed that Cu_2_O NPs were made in the process with representative PSD as reported using the dynamic light scattering method.

### 2.2. Effect of Rotation Speed on Particles Using Different Cu Precurors 

**Figure 5:**
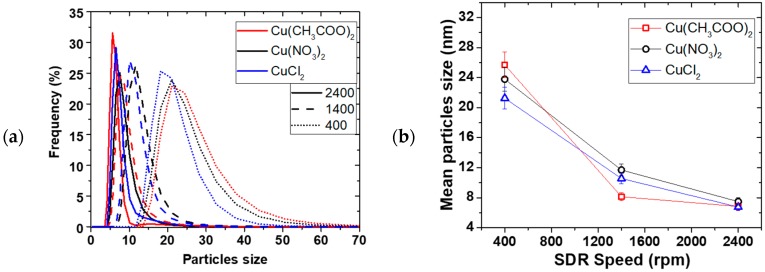
Effect of SDR disk speed on (**a**) PSD and (**b**) mean particle size of different Cu precursors, 3 mL/s of 0.01 M Cu^2+^ and 3 mL/s of 0.04 M NaBH_4_ (in 0.004 M NaOH).

Figure 5 shows the effect of the rotation speed on the particles size and PSD of different Cu precursors. Considering the error margin, the three precursors appear to show similar mean particles sizes with a slight distinction at 1400 rpm, where Cu(CH_3_COO)_2_ gave the smallest particle of 8.1 nm. Nevertheless, the mean particle size decreased and PSD narrowed with the increasing SDR rotation speed. Overall, the mean particle size ranged between 24 ± 2 to 6.8 ± 0.7 nm with increasing rotation speed. The trend was similar to what was observed and discussed earlier with the Cu(NO_3_)_2_ precursor. This suggested that the micromixing, residence time, and RTD of the SDR dictated the mean particles and PSD size as discussed earlier rather than the source of the Cu precursor. 

### 2.3. Effect of Reducing Agent and pH of the Reducing Agent

**Figure 6:**
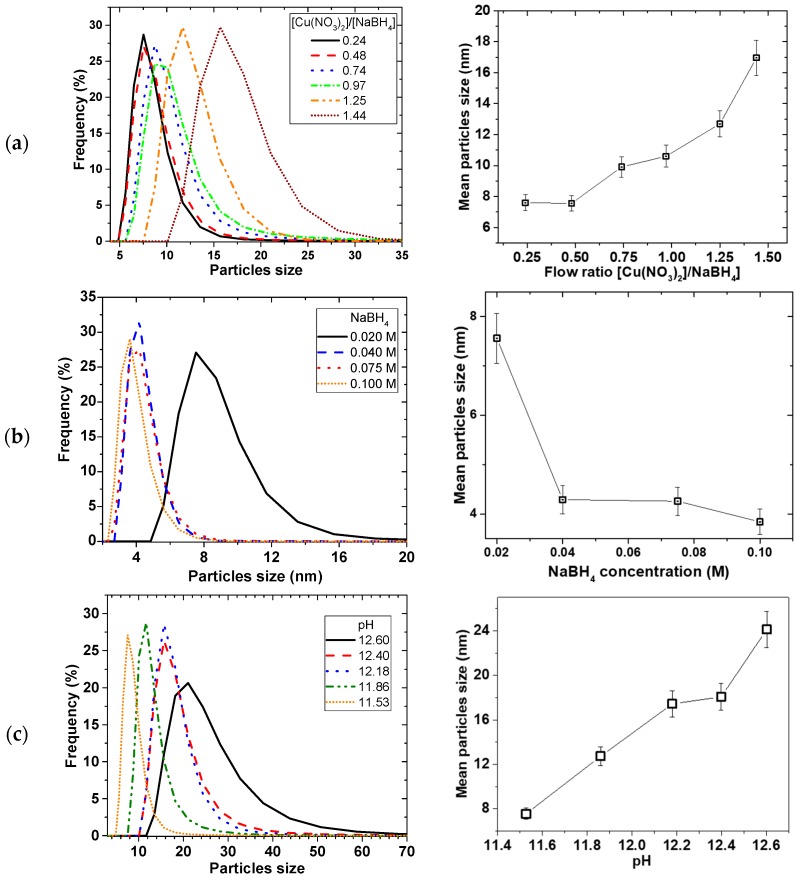
Effect of reducing agent on PSD (**left**) and mean particle size (**right**), (**a**) Effect of flow ratio, (**b**) Effect of NaBH_4_ concentration, (**c**) Effect of pH, (by varying only NaOH concentrations); 0.01 M Cu(NO_3_)_2_, 0.02 M NaBH_4_ (in 0.004 M NaOH), at 2400 rpm disk speed, 5 mL/s total flow rate.

Figure 6 shows the effect of the reducing agent on the particle size and PSD. As illustrated in Figure 6a, the mean particle size decreased with reducing flow ratio of Cu(NO_3_)_2_/NaBH_4_ (i.e., increasing NaBH_4_ flow at the expense of Cu(NO_3_)_2_ flow), from 17 ± 1 to 7.6 ± 0.5 nm then the sizes levelled off after ratio of 0.5. Figure 6b illustrates that increasing the concentration of NaBH_4_ at a constant Cu(NO_3_)_2_ concentration led to an initial decrease in mean particle sizes, and then levelled off after 0.04 M NaBH_4_ concentration. However, when Cu(NO_3_)_2_ and NaBH_4_ concentration and flow rates were kept constant and the amount of NaOH was varied, PSD widened and mean particle sizes increased linearly with pH (Figure 6c). 

Equation (5) showed that the Cu^2+^ reduction involves NaBH_4_ hydrolysis. Since NaBH_4_ is both soluble and reactive in water, NaOH was added to keep the NaBH_4_ in solution for the reduction process. It has been reported that the rate of NaBH_4_ hydrolysis increases with decreasing pH [25]. Ingersoll et al. [26] also reported that the amount of hydrolysis of NaBH_4_ can be enhanced catalytically, such that in the presence of NiCl_2_ and CoCl_2_ salts, the rate of hydrolysis decreased with increasing NaOH concentration. Considering the relatively short residence time for the reagents on the SDR, any factor that affects the reactivity and accessibility of the BH_4_^−^ can have consequence on the precipitation reaction to affect the particle size and PSD. This was evident when particle size linearly increased with pH (Figure 6c) as the amount of OH^−^ increased supressing the reactivity of the NaBH_4_.

Furthermore, the stoichiometry of the reaction (Equation (5)) requires that the amount of NaBH_4_ should be more than the Cu(NO_3_)_2_ to reduce all the Cu precursor towards precipitation. When NaBH_4_ becomes the limiting reagent, the amount of reactive NaBH_4_ readily available for the Cu^2+^ reduction is decreased, thereby increasing the time to attain homogeneity of the mixture. Subsequently, this leads to less uniform nuclei formation and there is delay in adequate micromixing, leading to patchy growth. Considering the short retention time involved in the process, wider PSD with bigger particles occurs as the amount of NaBH_4_ decreased. On the other hand, when the amount of NaBH_4_ increases to a certain maximum (for e.g., 0.04 M BH_4_^−^:0.01 M Cu^2+^), the relative increase in reactive NaBH_4_ reaches a saturation point and all the Cu reacts with NaBH_4_ in a shorter time such that excess NaBH_4_ will have no further effect. 

### 2.4. Scaling up Cu NP Production and Methanol Synthesis

**Figure 7:**
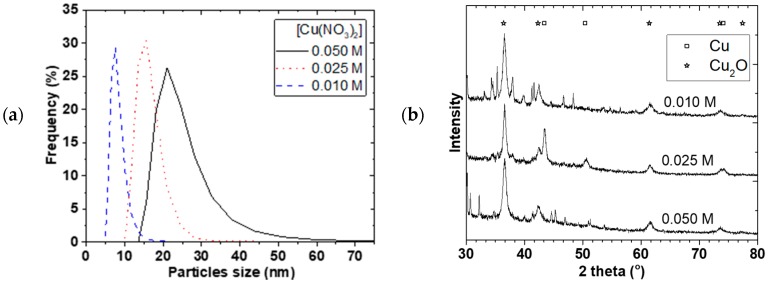

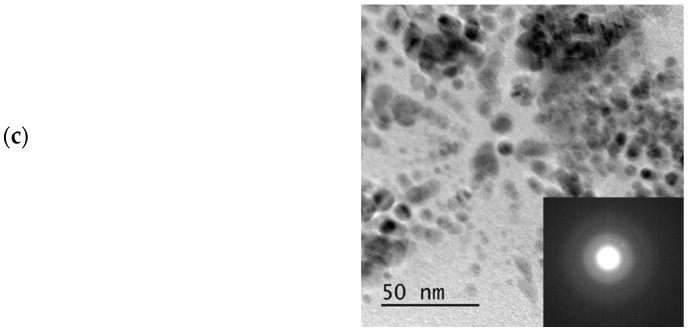
Effect of Cu(NO_3_)_2_ concentration on NP, (**a**) Effect of concentration on PDS, (**b**) XRD of the dried samples, (**c**) TEM image and diffraction of the dried 0.01 M sample; at 2400 rpm, 1Cu^2+^:4BH_4_^−^ concentration, 9 mL/s flow.

To apply the Cu NP as catalyst for MeOH synthesis, a larger amount of NP production was required over a longer continuous processing time. Firstly, 1 L of different starting Cu(NO_3_)_2_ concentrations were used at a 2400 rpm rotation speed and 1Cu(NO_3_)_2_:2NaBH_4_ flow rate. Figure 7a,b shows the PSD and X-ray diffraction for 0.01, 0.025, and 0.050 M starting Cu(NO_3_)_2_ concentrations. The mean particle sizes increased from 7.6 ± 0.5 to 22 ± 2 nm and the PSD widened when the Cu(NO_3_)_2_ concentration was increased. Separation of the NP from solution was challenging and we resorted to oven drying at 90 °C. Mainly Cu_2_O and Cu crystallite (+ some NaNO_3_ phase) were observed and the crystallite sizes slightly increased from 8.6 ± 0.2 to 10.8 ± 0.3 nm with increasing concentration even though the mean particle sizes were generally larger. A similar observation was made by Chang et al. [17] where mean particle size increased from 48.3 to 93.0 nm when Cu(SO_4_)_2_ concentration was increased from 0.01 to 0.40 M. As concentration increased, the probability of nuclei colliding with each other increased leading to agglomeration and larger particles size as well as wider PSD. 

Figure 7c shows the TEM image and electron diffraction of the Cu NP made at 0.01 M Cu(NO_3_)_2_ starting concentration. The spherical shaped polycrystalline Cu_2_O observed in the TEM image, which showed particle sizes around 10 nm, confirmed that the method can be scaled-up. The challenge however, is the separation of the particles from the starch, as the starch gelatine used was no more soluble in water. Given that the resulting slurry was colloidal in nature, it was difficult to use a centrifuge to isolate the NPs. As a result, the slurry was dried at 90 °C, which could lead to possible increase in agglomeration of particles. If the reaction were carried out in solvent of interest for further processes or the catalysis in our case, then there would not be any need for separation or drying. However, our equipment at the time of this study was not materially compatible with ether solvents and we resorted to the use of water as a solvent. 

**Figure 8:**
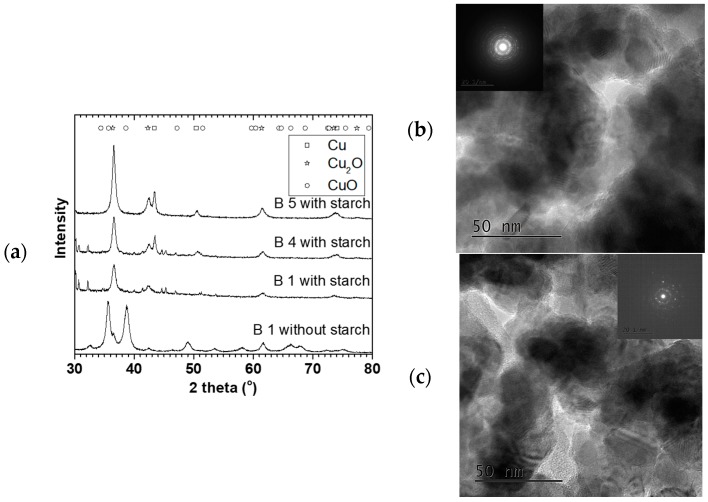
Scale-up Cu NP for MeOH synthesis, S = in starch, NS = No starch, (**a**) XRD, (**b**) TEM image of B 4S, (**c**) TEM image of B 5S.

**Figure 9:**
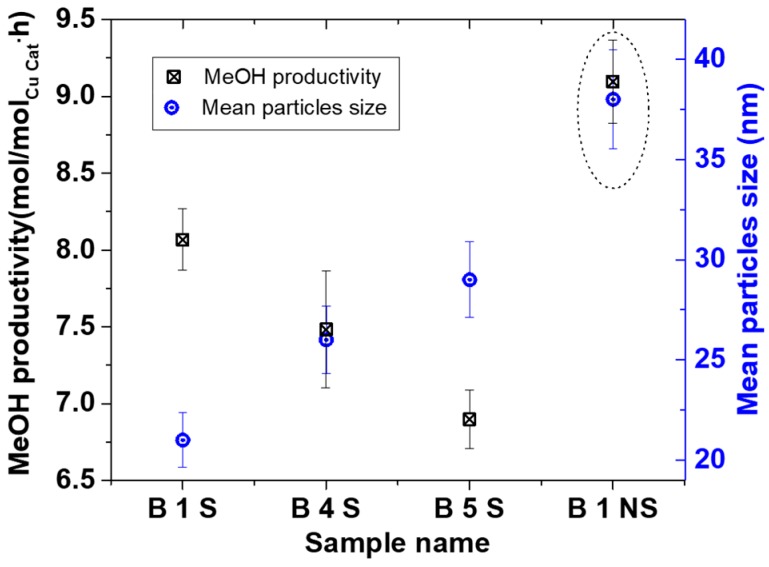
Low temperature MeOH synthesis of scale-up Cu NP; 2H_2_/CO = 20 bar, THF solvent = 30 mL, for MeOH synthesis.

Furthermore, based on our knowledge of controlling Cu NP from the above, three tailored Cu NP with different mean particle sizes collected in starch were used for MeOH synthesis. Figure 8 shows XRD and TEM characterization for some 90 °C oven dried samples. These particles were spherical and polycrystalline, with mainly Cu_2_O and Cu^0^ phases. The crystallite sizes were 8.6 ± 0.5, 9.0 ± 0.6, and 9.5 ± 0.7 nm with 21 ± 1, 26 ± 2 and 29 ± 2 nm particles sizes for B1 S, B 4 S, and B 5 S respectively (S = with starch, see Figure 9). In addition, for comparison, B1 was repeated without starch (NS), where CuO was the predominant phase with 9.4 ± 0.7 nm crystallite and 38 ± 2 nm mean particle sizes. This suggested that aside from reducing agglomeration of the particles, the starch also reduced the amount of surface oxidation of the resulting NPs.

Syngas conversion over the selected samples ranged from about 50% to 70% conversion per batch, which is around the same range achieved in other Cu-alkoxide systems [8,27]. However, for comparison reasons, the amount of MeOH produced per amount of Cu (in mol/(mol·h)) is presented. Figure 9 shows the MeOH productivity compared with particle sizes. The MeOH productivity generally increased with decreasing particles sizes. This has already been attributed to the increase in total surface area as particle sizes decreased [7,10]. 

The B1 NS sample showed a higher MeOH productivity than all the smaller particles collected in the starch. This sample differed from the other starch containing specimens by particle size and Cu phases present. Previous results have shown that in the presence of 20 bar CO/2H_2_ at 100 °C, reduction of Cu^2+^ is rapid [10,28], and that the active Cu phase for the MeOH synthesis is assumed to be in the Cu^+^/Cu° oxidation state [29]. This implies that it is not likely that the Cu phase in the B 1 NS catalyst contributed to the difference in the activity but rather the absence of starch. Hence, the lower activity of the catalyst samples containing starch could be due to mass transfer limitations of the substrate in accessing the surface of the Cu NP. This is in contrast to the B 1 NS catalyst where no starch is present on the surface. Nevertheless, the scaled-up Cu materials made with the SDR were active for MeOH synthesis either with or without starch present and can be further explored for optimization.

## 3. Materials and Methods 

### 3.1. Spinning Disk Reactor

**Figure 10:**
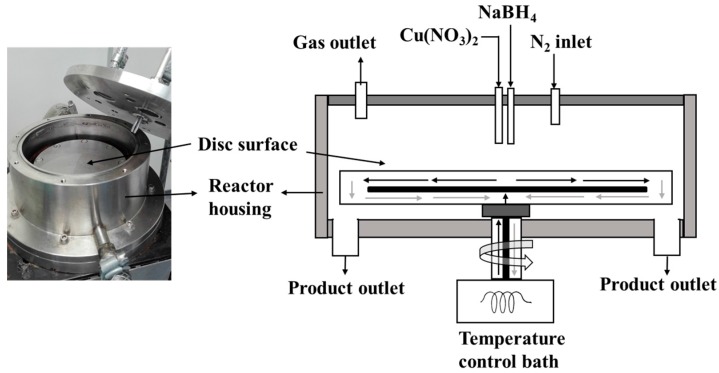
Scheme of SDR set-up used in making Cu NP.

The set-up used in this work is shown in the schematic diagram in Figure 10 similar to the one described elsewhere [13,14]. The 10 cm diameter smooth surfaced stainless steel disk was driven by a 125 W electric motor, coupled with a digitally controlled rotating disk. A temperature controlled water-bath was circulated beneath the disk to ensure constant disk temperature (at 25 °C). Cu(NO_3_)_2_ solution in one line and NaBH_4_ dissolved in NaOH solution in another line were fed onto the centre of the spinning disk. A Watson Marlow 323 peristaltic pump coupled with a dampener at the discharge end was used to control smooth flow of the two reagents. Each feed tube made of Viton, with 3.2 mm hole ends was set at a distance 6 mm perpendicular to the centre of the spinning disk. The reaction was carried out in a N_2_ blanket to minimize direct contact of the reaction with air.

As shown in the Equation (5), Cu particles were precipitated by borohydride reduction. Typically, 0.01–0.05 M standard solutions of Cu(NO_3_)_2_ was reacted with 0.02–0.20 M NaBH_4_ dissolved in about 17 *w*/*w* % NaOH. Since NaBH_4_ is both soluble and reactive in water, adding the NaOH was necessary to keep NaBH_4_ in solution. To avoid agglomeration and settling of the particles after collection from the SDR, samples were collected in starch as has been used elsewhere [16]. Thus, the resulting product was collected in 1 wt % starch gelatine solution. The starch gelatine was prepared by dissolving 10 g starch in 1000 mL water heated to 90 °C. The influence of starch on the particle size distribution for a period is shown in the supplementary material Figure S1. 

### 3.2. Characterization of Cu Nanoparticles and Methanol Synthesis

Mean particle sizes and particles size distributions were analysed using dynamic light scattering (Malvern instrument, Model HPPS) with a He-Ne laser as light source (λ = 633 nm) and measured at 25 °C. Samples to be used for MeOH synthesis were dried at 90 °C for further characterization. A Bruker D8 discover powder diffractometer using Cu K-α-1 radiation (λ = 1.5 Å) selected by a Ge (111) Johansen monochromator and a Lynxeye detector were used. The diffractogram was measured at 0.025° steps per second. Total Pattern Analysis Solution (TOPAS) software was employed for quantitative Rietveld analysis of the diffractogram. This software operates by fitting a theoretical diffraction pattern to a measured diffraction pattern using non-linear least square algorithms [30]. The SEM imaging was performed using SU8230 ultra-high resolution cold-field emission SEM from Hitachi. The TEM imaging was performed with a Joel 2100F instrument. Diluted samples were dispersed in an ultrasound bath for 30 min and then deposited onto a carbon film on a copper grid. 

MeOH synthesis was done similar to the process described in [10,31] in a 200 mL stainless high pressure hpm-020 autoclave batch reactor (Premex Reactor AG), equipped with a pressure sensor and thermocouple inserted. Weighed Cu NP and sodium methoxide (NaCHO_3_) were added to 50 mL diglyme placed in the reactor. The reactor was charged to about 20 bar syngas (1CO:2H_2_), then stirred at 3000 rpm and heated to 100 °C. The cooled liquid products were analysed using Agilent 7890 A GC with Agilent 7683B autosampler coupled with Agilent 5975 mass spectrometer detector (MSD). A CARBOWAX 007 series 20 M column with dimensions 60 m × 320 µm × 1.2 µm was used and programmed at 15 °C/min temperature ramp from 40 °C to 200 °C and held at 200 °C for 3 min, at 0.47 bar (6.8 psi) constant pressure. 0.54 mg heptane was as an internal standard.

Dry powdery sample was used for most of the characterization (XRD and TEM) and MeOH synthesis. Given that the collected sample from the SDR was colloidal in nature when collected in starch, it was difficult to use centrifuge for isolating the Cu NP. Slurry samples in smaller proportions were oven dried at 90 °C overnight. The dry samples were used for the MeOH synthesis without purification. MeOH productivity was calculated as shown in Equation (6).

(6) $$Productivity=\frac{amount\;of\;MeOH\;\left(\mathrm{mol}\right)}{amount\;of\;Cu_{catalyst}\;\left(\mathrm{mol}\right)\times reaction\;time\;\left(h\right)}$$

## 4. Conclusions

The SDR was used for making varying copper NP sizes based on copper borohydride reduction reaction. The Cu NP sizes and distributions were varied by changing physical and chemical parameters involved in the precipitation reaction. Particle size distribution narrowed with a corresponding decrease in particle size when micro-mixing time was shortened by, for example, increasing SDR rotation speed and total flow rates. Particle sizes in the range of 3 to 55 nm were obtained, which upon oven drying at 70 or 90 °C showed predominantly polycrystalline Cu_2_O and Cu phases. The advantage of the current technique is that it provides a faster approach to fine tuning Cu NP sizes for MeOH synthesis by varying physical parameters but using the same chemical recipe. The NPs were tested to be active for MeOH synthesis at low temperature (100 °C) and MeOH productivity increased with decreasing particle sizes. 

## Supplementary Materials

The following are available online at www.mdpi.com/1996-1944/11/1/154/s1, Figure S1: Effect of starch concentration on PSD.
