# Supplementary material for: Tailoring Cu Nanoparticle Catalyst for Methanol Synthesis Using the Spinning Disk Reactor
Source: Materials (Basel). 2018 Jan 17;11(1):154. doi: 10.3390/ma11010154 (PMC5793652; doi:10.3390/ma11010154)
Supplement: Supplementary file 1 [file materials-11-00154-s001.pdf]

Tailoring Cu nanoparticles catalyst for methanol synthesis using the spinning disk reactor

Christian Ahoba-Sam[1], Kamelia V.K. Boodhoo[2], Unni Olsbye[3] and Klaus-Joachim Jens[1,*]

[1] Department of Process, Energy and Environmental Technology, University College of Southeast Norway, Kjølnes Ring 56, 3918 Porsgrunn, Norway; christian.ahoba-sam@usn.no

[2] School of Engineering, Merz Court, Newcastle University, Newcastle Upon Tyne NE1 7RU, UK; kamelia.boodhoo@newcastle.ac.uk

[3] Department of Chemistry, University of Oslo, P.O. Box 1033, Blindern, N-0315, Oslo, Norway; unni.olsbye@kjemi.uio.no

[1*] Correspondence: Klaus.J.Jens@usn.no; Tel.: +47 35575193

Supplementary data

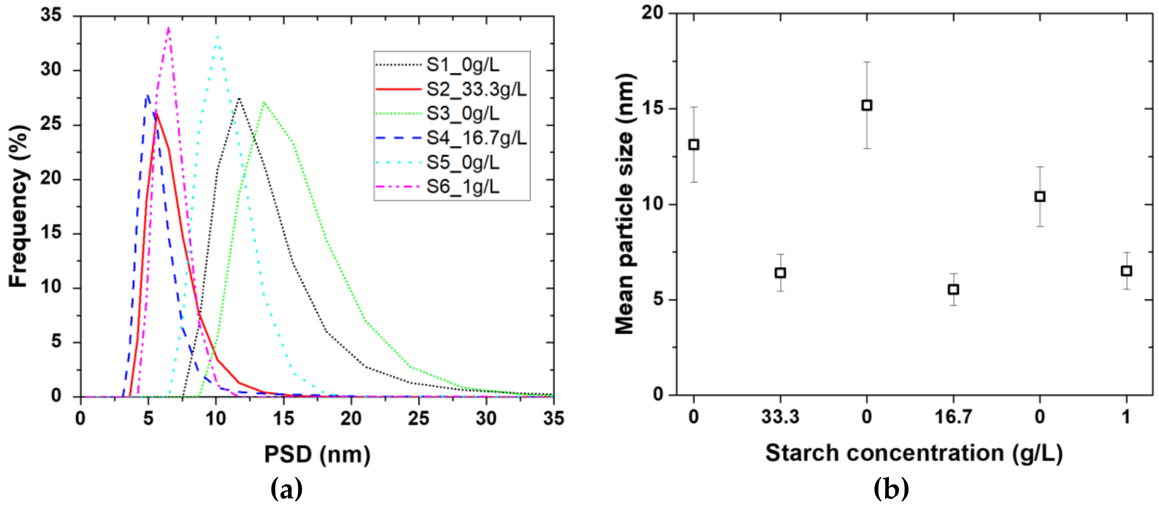

Figure S1. Effect of starch concentration on PSD (a) and mean particles size (b), 0.01 M $Cu(NO_3)_2$/0.02 M $NaBH_4$, flow ratio=2, flow rate=5.5 ml/s disk speed= 2400 rpm

In order to keep the Cu nanoparticles from agglomerating, corn-starch was dissolved in 90 ^{o}C hot water to form starch gelatine. Fig S1 shows the effect of varying starch concentration on the particles size after 1 day. When no starch was added, wider particles size distribution (PSD) was observed, with varying mean particles sizes, ranging from 10 to 15 nm. However when the particles were collected in starch gelatine, narrow PSD was observed and the mean particles size was about 6±1 nm. Moreover, no significant difference in both the PSD and the mean particle sizes were observed from the 1 to 33.3 1 g/L starch. As a result, the Cu nanoparticles made with the SDR were collected directly into 10 ml of 1 wt % (10g/L) starch gelatine.
